# Delayed Time to Cryptosporidiosis in Bangladeshi Children is Associated with Greater Fecal IgA against Two Sporozoite-Expressed Antigens

**DOI:** 10.4269/ajtmh.20-0657

**Published:** 2020-10-19

**Authors:** Kevin L. Steiner, Mamun Kabir, Biplob Hossain, Carol A. Gilchrist, Jennie Z. Ma, Tahmeed Ahmed, Abu S. G. Faruque, Rashidul Haque, William A. Petri

**Affiliations:** 1 Division of Infectious Diseases and International Health, Department of Medicine, University of Virginia, Charlottesville, Virginia;; 2 International Centre for Diarrhoeal Disease Research, Bangladesh (icddr,b), Dhaka, Bangladesh;; 3 Division of Biostatistics, Department of Public Health Sciences, School of Medicine, University of Virginia, Charlottesville, Virginia

## Abstract

Cryptosporidiosis is common in early childhood, and both diarrheal and subclinical infections are associated with adverse developmental outcomes. Improved therapeutic medications may help reduce the burden of cryptosporidial diarrhea; however, an effective vaccine would be better able to prevent the detrimental impact of both diarrheal and subclinical disease. A more complete understanding of naturally occurring immunity may further inform strategies to develop an effective vaccine. In this prospective cohort study of Bangladeshi children, greater fecal IgA at 12 months, but not plasma IgG, directed against two sporozoite-expressed, immunodominant and vaccine candidate antigens was associated with delayed time to subsequent cryptosporidiosis to 3 years of life. These findings extend prior work and further support the role of mucosal antibody responses in naturally developing protective immunity to *Cryptosporidium*.

Cryptosporidiosis, caused by intracellular protozoan parasites of the genus *Cryptosporidium*, is a globally common infection with long-term adverse developmental consequences.^1–3^ The parasite is a leading cause of childhood diarrheal morbidity and mortality in many low- and middle-income countries; however, there is increasing recognition that subclinical (non-diarrheal) cryptosporidiosis is common and associated with impaired growth.^2–4^ Although several new therapeutics are under development, nitazoxanide is currently the only U.S. Food and Drug Administration–approved treatment for cryptosporidial diarrhea, and its efficacy in young children and other vulnerable populations is limited.^5^ Addressing the burden of cryptosporidial diarrhea through better therapeutics is crucial; however, even if proven clinically effective, these therapies would rely on infected children presenting to medical care for diagnosis and treatment. In the absence of diarrhea, one may expect most subclinical infections to remain untreated with subsequent developmental consequences remaining unchecked. Development of an effective vaccine remains an important and appealing approach to better mitigate the full burden of the parasite. Greater understanding of naturally occurring immunity to the parasite is important to better inform vaccine development strategies. Cell-mediated immune responses, particularly CD4^+^ T-cell production of interferon gamma (IFN-γ), are critical to clearance of established *Cryptosporidium* infection.^6,7^ We previously showed that greater fecal IgA directed against a sporozoite-expressed antigen measured at 12 months of age was associated with delayed time to subsequent cryptosporidiosis over the subsequent year, suggesting that antibody-mediated immune responses may play a role in preventative immunity.^8^ Whether this protective association persisted beyond age 2 years and whether it was unique to the studied sporozoite-expressed antigen (Cp23) or whether responses to other sporozoite-expressed antigens may similarly be protective remained uncertain.

To address these questions, we again leveraged an ongoing, prospective birth cohort study located in Mirpur, Dhaka, Bangladesh, and in which active surveillance of children, for whom informed written consent was provided by a parent or guardian, was continued through 3 years of life, as previously described.^3^ The Ethics and Research Review Committee at the icddr,b approved this study, and a reliance agreement was granted by the Institutional Review Board of the University of Virginia. Diagnosis of cryptosporidiosis was based on detection by real-time PCR of stool samples which were collected monthly and during diarrheal episodes (≥ 3 loose stools in 24 hours).^3^ Plasma was obtained from children at 12 months of age. The sporozoite-expressed antigens Cp17 and Cp23 lacking the glutathione *S*-transferase expression tag were prepared, as previously described.^9^ These antigens were chosen because both are surface-expressed during the infective sporozoite parasite stage, have been previously shown to induce both antibody- and cell-mediated immune responses, and are considered potential vaccine candidates.^9–12^ Anti-Cp17 and anti-Cp23 plasma IgG and fecal IgA were measured using ELISA.^8,9^ Subjects were divided into the upper and lower 50th percentiles for each antigen–antibody pairing. Survival probabilities for time to first *Cryptosporidium* PCR-positive stool from 12–36 months of age were estimated with the Kaplan–Meier method for both plasma IgG and fecal IgA for Cp17 and Cp23, respectively. Univariate Cox regression of time to subsequent cryptosporidiosis was performed for demographic, socioeconomic, and anthropometric variables (Supplemental Table 1). Variables for which *P* < 0.1 were then included in multivariable Cox regression analysis. Analyses were performed using R version 4.0.0 with package “survival” version 3.1–12 (R Foundation for Statistical Computing, Vienna, Austria) with function “coxph.”

Enrolled infants were born between July 2014 and April 2016 and were subsequently prospectively followed to age 3 years. Stool and plasma samples obtained at 12 months of age were available for 442 children. Details of gender, household size and income, duration of excluding breastfeeding, water source and treatment, and length-for-age *z* score (LAZ) at 12 months have previously been reported.^8^ As previously described, 126 children (28.5%) had detectable cryptosporidiosis in the first year of life. By age, 3, 340 children (81.9%) had a PCR-detected *Cryptosporidium* infection; 27 children were included in the analysis but censored (migrated out of the study area or a parent/guardian elected to withdraw from the study) before age 3 years without preceding *Cryptosporidium* infection.^8^

There was no difference observed in cryptosporidiosis-free survival through age 3 years of life between children in the upper or lower 50th percentiles for plasma IgG measured at 12 months of age and directed against either Cp23 or Cp17 (Figure 1A and C). However, children in the upper 50th percentile of fecal IgA measured at 12 months and directed against either Cp23 or Cp17 were more likely to be subsequently cryptosporidiosis-free through age 3 years of life than children in the lower 50th percentile (Figure 1B and D; *P* = 0.0034 and 0.031 for Cp23 and Cp17, respectively).

**Figure 1.**
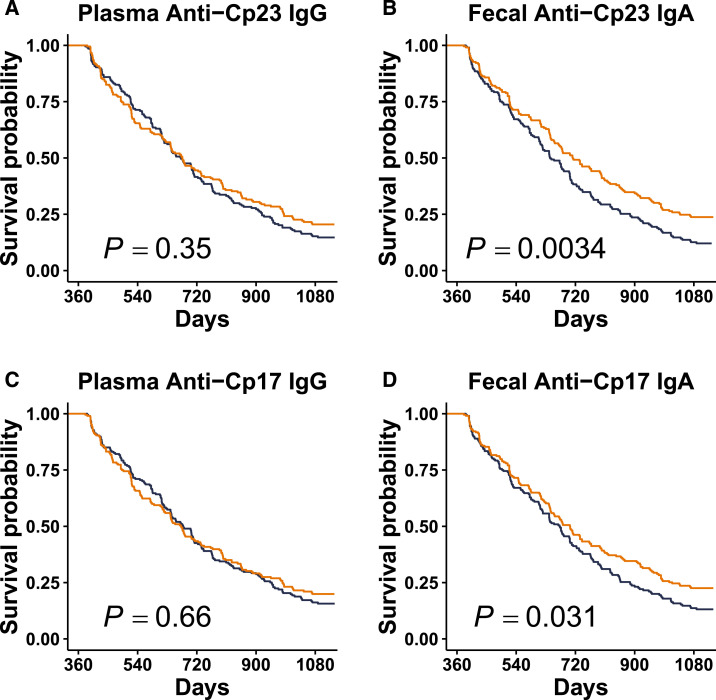
Kaplan–Meier curves showing probability of survival free of *Cryptosporidium* species from age 1 to 3 years among infants (*n* = 442) stratified by quantity of anti-Cp23 or anti-Cp17 immunoglobulin, respectively. (**A** and **C**) No difference in subsequent cryptosporidiosis-free survival between children in the upper 50th (orange) and lower 50th percentile (blue) of plasma IgG measured at 1 year of life against Cp23 or Cp17, respectively. (**B** and **D**) Children in the upper 50th percentile (orange) of fecal IgA measured at 1 year of life against Cp23 or Cp17, respectively, had statistically significantly longer subsequent cryptosporidiosis-free survival than children in the lower 50th percentile (blue). The *y* axes represent the survival probability free from infection, and the *x* axes are survival time in days, from 1 to 3 years of life. *P* values are estimated by using the log-rank test.

Univariate Cox regression analysis was performed for potentially important covariables including anti-Cp23 and anti-Cp17 antibody responses and demographic, socioeconomic, and anthropomorphic factors (Supplemental Table 1). As we have previously shown seasonal and yearly differences in the incidence of cryptosporidiosis in Bangladesh, the month and year of birth were also included (Supplemental Table 1).^13^ In addition to fecal IgA in the upper 50th percentile against Cp23 and Cp17, only monthly income and LAZ at 12 months had *P* < 0.1. Multivariable Cox regression analysis to time to subsequent cryptosporidiosis was performed using these variables with *P* < 0.1; because of correlation of fecal Cp23 and Cp17 IgA only fecal anti-Cp23 IgA was included. In multivariable analysis, only fecal Cp23 IgA in the upper 50th percentile remained statistically significant with a reduction in the hazard ratio of 21% (95% CI: 1–36%; *P* = 0.04; Table 1).

**Table 1:** Multivariable Cox regression indicating a statistically significant decrease in the hazard ratio of subsequent *Cryptosporidium* infection through 3 years of life for children in the upper 50th percentile of fecal anti-Cp23 IgA

Variable	Hazard ratio (95% CI)	*P*-value
Fecal anti-Cp23 IgA in the upper 50th percentile	0.79 (0.64–0.99)	0.04
Monthly income (in thousands Bangladeshi Taka)	0.99 (0.99–1.00)	0.17
Length-for-age *z* score at 12 months	0.91 (0.81–1.01)	0.09

Variables included in multivariable analysis were selected if univariate *P* < 0.1 (see Supplemental Table 1). As fecal anti-Cp23 IgA in the upper 50th percentile was highly correlated with fecal anti-Cp17 IgA in the upper 50th percentile, only fecal anti-Cp23 IgA was included in multivariable analysis.

These findings extend our previous observations of association of greater anti-*Cryptosporidium* fecal IgA (but not plasma IgG) with delayed time to subsequent cryptosporidiosis in two important ways. First, we show that the association of greater fecal IgA directed against well-described and immunogenic sporozoite-expressed antigens at 12 months of age with subsequent protection from infection persists (though wanes) through 3 years of life. Importantly, this association of greater fecal anti-*Cryptosporidium* IgA with delayed subsequent cryptosporidiosis persisted and remained statistically significant in stepwise multivariable analysis; other demographic, socioeconomic, anthropometric, and month and year of birth were not associated with protection. Second, we show that this protective effect of specific fecal IgA may be seen not only with Cp23 but also with a second sporozoite-expressed antigen, Cp17, which has been used for serologic prevalence studies and evaluated as a potential vaccine candidate.^6,14,15^ In addition, we again show that greater plasma IgG directed against these sporozoite-expressed antigens confers no protective benefit, similar to our previous findings and consistent with those of a prior birth cohort study in India.^8,16^

Strengths of this study include the size, prospective birth cohort design, and setting in an area of naturally occurring cryptosporidiosis. Measuring antibody responses against two sporozoite-expressed, immunodominant, and vaccine candidate antigens is an additional strength. A major limitation of the primary outcome (time to subsequent cryptosporidiosis) is the frequency of testing for non-diarrheal cryptosporidiosis which occurred monthly. There is potential for “missed” subclinical infections that occur and are cleared between monthly surveillance stool samples. In addition, it is likely that fecal anti-*Cryptosporidium* IgA levels are dynamic and may change over time; this study measured fecal IgA at a single time point (12 months). Further studies are in process to better characterize the dynamics of anti-Cp23 and anti-Cp17 fecal IgA over time. For some children, it is possible the measured IgA in stool was acquired via maternal breast milk; however, as we previously reported, mean exclusive breastfeeding was approximately 3.5 months and days of exclusive breastfeeding was not a significant variable in the Cox analysis.^8^ We therefore consider maternally derived anti-*Cryptosporidium* IgA unlikely to be a major contributor to our findings.

There is increasing evidence for development of naturally occurring immunity to *Cryptosporidium*, which combined with the well-described adverse effect of even subclinical early childhood cryptosporidiosis, provides increased support for further vaccine development efforts.^3,17^ Neither this nor any other prior study has directly demonstrated a protective effect in humans of immune responses to either Cp23 or Cp17; nevertheless, our findings further support the hypothesis that antibody-mediated immunity may serve an import role in naturally occurring protective immunity. Other target antigens have also been described and tested in animal models including circumsporozoite-like antigen, SA35, and SA40, as recently reviewed.^6,18^ Further efforts are needed to identify which antigens, alone or in combination, and adjuvants may be most efficacious in inducing protective immunity. One potential model includes high levels of anti-*Cryptosporidium* IgA at the mucosal surface and within the intestinal lumen capable of preventing (or the reducing frequency of) successful invasion of intestinal epithelial cells (IECs). In the event of successful IEC infection, cell-mediated responses, including those driven by IFN-γ–producing CD4^+^ T cells, become critical for clearing immunity and eradication of established infection.^6,10,19^ Future vaccine development approaches ideally would robustly induce both mucosal (i.e., secretory IgA) antibody-mediated and cell-mediated responses.

## Supplemental table

Supplemental materials
