# Supplementary material for: Delayed Time to Cryptosporidiosis in Bangladeshi Children is Associated with Greater Fecal IgA against Two Sporozoite-Expressed Antigens
Source: Am J Trop Med Hyg. 2020 Oct 19;104(1):229–32. doi: 10.4269/ajtmh.20-0657 (PMC7790099; doi:10.4269/ajtmh.20-0657)
Supplement: Supplementary file 1 [file tpmd200657.SD1.pdf]

Supplemental Table 1: Univariate Cox regression analysis for demographic, socioeconomic, and anthropomorphic (maternal and child) variables. Variables with $P < 0.1$ were subsequently included in multivariable Cox regression analysis.

**Variable**	**Hazard ratio (95% confidence interval)**	***P***
Fecal anti-Cp23 IgA in upper 50th percentile	0.73 (0.59-0.90)	0.003
Fecal anti-Cp17 IgA in upper 50th percentile	0.79 (0.64-0.98)	0.03
Plasma anti-Cp23 IgG in upper 50th percentile	0.9 (0.73-1.1)	0.35
Plasma anti-Cp17 IgG in upper 50th percentile	0.95 (0.77-1.2)	0.66
Sex (male reference)	0.91 (0.74-1.1)	0.38
Mother BMI	0.99 (0.96-1)	0.34
Mother Age	0.99 (0.97-1)	0.35
Household size	0.97 (0.93-1)	0.21
Monthly income (in 1000s Bangladeshi Taka)	0.99 (0.98-1)	0.09
Exclusive breastfeeding days	1 (1-1)	0.87
Gestational age (weeks)	0.99 (0.93-1.1)	0.74
Length-for-age *z* score (LAZ) at 12 months	0.89 (0.8-0.99)	0.03
Month of birth	0.99 (0.96-1)	0.42
Year of birth	1 (0.84-1.2)	0.97
